# The complete chloroplast genome sequence of the medicinally important plant *Serissa serissoides* (DC.) Druce and phylogenetic analysis

**DOI:** 10.1080/23802359.2026.2706902

**Published:** 2026-07-27

**Authors:** Xianglan Liang, Qiuxiang Luo, Song Guo

**Affiliations:** a College of Smart Agriculture (College of Internet of Things Engineering), Guangxi Science & Technology Normal University, Guangxi Laibin, China; b School of Chemistry and Chemical Engineering, Guangdong Pharmaceutical University, Guangdong, Guangzhou, China; c Yao Ethnic Medicine Hospital of Jinxiu Yao Autonomous County, Guangxi Laibin, China

**Keywords:** *Serissa serissoides*, chloroplast genome, phylogenetic analysis

## Abstract

*Serissa serissoides* (DC.) Druce, a well-known traditional Yao medicinal plant belonging to the genus *Serissa*, was investigated in this study. We firstly characterized its complete chloroplast genome, which is 154,653 bp in size and possesses a typical quadripartite structure consisting of an 84,139 bp LSC region, a 17,060 bp SSC region and a pair of 26,727 bp IR regions. In total, 130 functional genes were annotated, including 84 protein-coding, 38 tRNA and 8 rRNA genes. Phylogenetic analysis demonstrated that *S. serissoides* is phylogenetically close to species of the genus *Leptodermis*.

## 1. Introduction

*Serissa serissoides* (DC.) Druce, 1917, is a member of the genus *Serissa* (family Rubiaceae, order Gentianales) and is widely distributed in China. The genus *Serissa* comprises only a few species, mainly distributed in subtropical and tropical regions of Asia. *S. serissoides* is widely distributed in southern China, and typically grows in hillsides, sparse forests, and riverbanks. In traditional Chinese medicine, *S. serissoides* has long been used to treat liver-related diseases, including hepatitis and hepatocirrhosis (Zhao et al. 2011). In Yao traditional medicine, it is known as “Ji Jing Feng” and is regarded as a classic medicinal resource, traditionally used to dispel wind, remove dampness, clear heat, and detoxify. Phytochemical studies have revealed that *S. serissoides* contains various bioactive compounds, including lignans, terpenes, volatile oils, and polysaccharides (Zhao et al. 2011). Pharmacological research demonstrates that its water extract inhibits the secretion of HBsAg and HBeAg in the HepG 2.2.15 cell line and protects against liver injuries induced by carbon tetrachloride, acetaminophen, and thioacetamide (Zhang and Sun 2006). The extract of *S. serissoides* also increased the avoirdupois and thymus gland index of the tested mice and showed antimicrobial activities (Li et al. 2007; Zhao et al. 2011).

Although *S. serissoides* has important clinical applications, the complete chloroplast genome has not been reported. The aim of this study is to provide the complete chloroplast genome of *S. serissoides* to bridge this gap and provide the basis for further study of genetic traits of this plant.

## 2. Materials and methods

**Figure 1.**
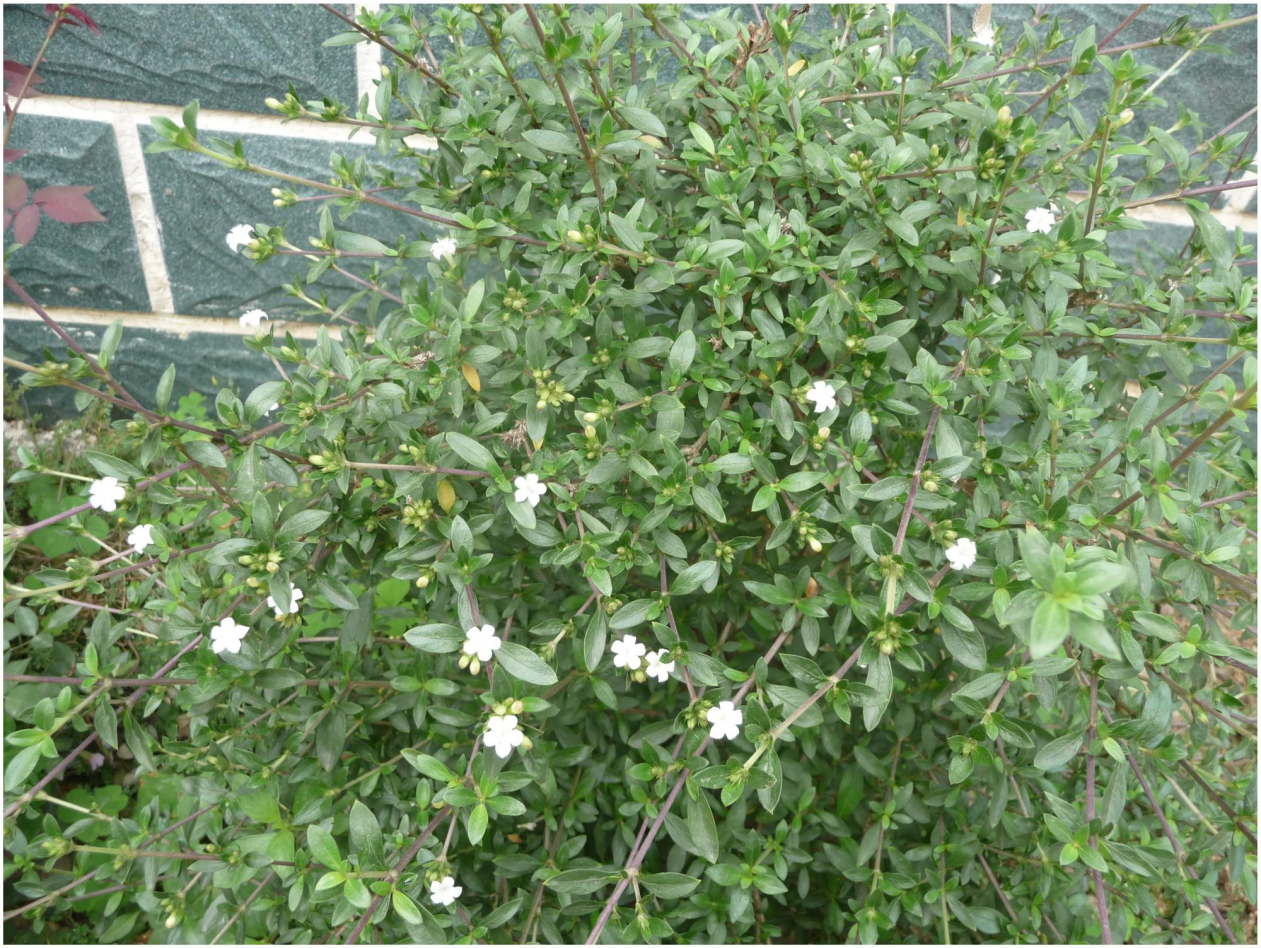
The pictures of *S. serissoides* (DC.) Druce., photographed by Qiuxiang Luo (114486349@qq.com) in Dayao Mountain, Jinxiu Yao Autonomous County, Laibin City, Guangxi Zhuang Autonomous Region, China (N:24°9’22.100”, E:110°7’12.943”). Morphological characteristics: small shrub, usually up to 1 m tall; young branches are covered with soft hairs; leaves are opposite, thin in texture and vary greatly in shape, with acute to slightly blunt tips; flowers are white and clustered at the tops of small branches; bracts are membranous and the calyx has ciliate margins; corollas are oblong-lanceolate, pointed at the tips, with anthers enclosed; drupes are nearly spherical. The flowering period is from April to June.

Fresh and healthy leaves of *S. serissoides* were collected from a wild individual in Jinxiu Yao Autonomous County, Guangxi Province, China (N:24°9’22.100”, E:110°7’12.943”) (Figure 1). A voucher specimen was deposited in Room 404, at College of Biology and Food Engnineering, Guangxi Science & Technology Normal University (Song Guo, email: guosong0804@163.com) underthe voucher number JJF202006. Song Guo identified the plant specimen (Figure 1).

Total genomic DNA was extracted from fresh leaves by using a DNeasy Plant Mini Kit (QIAGEN, Hilden, Germany) following the manufacturer’s instructions. High-quality DNA was submitted to Biomarker Technologies (Beijing, China) for genomic library construction and sequencing on the Illumina HiSeq platform (Illumina, San Diego, CA, USA). Approximately 4.4 GB of raw sequencing data were generated. Raw sequencing reads were quality-filtered using Fastp v0.2 (Chen 2023) to remove adapter sequences and low-quality reads prior to chloroplast genome assembly. The chloroplast genome of *S. serissoides* was assembled using GetOrganelle v1.7.7.1 (Jin et al. 2020) with default parameters. The assembled chloroplast genome was mapped using BWA (Li 2013), and coverage depth was calculated with Samtools (Danecek et al. 2021). The chloroplast genome was annotated using both CPGAVAS2 (Shi et al. 2019) and GeSeq tools (Tillich et al. 2017). The annotated genomic sequence has been submitted to GenBank with the accession number: PP621515.2. A circular map of the chloroplast genome and visualizations of cis-splicing and trans-splicing genes were generated using the online tool CPGview (Liu et al. 2023).

19 additional species and *Rauvolfia serpentina* (NC_047244.1) as outgroup, which used for phylogenetic analysis. The shared protein-coding genes (PCGs) of the chloroplast genomes were identified using BLASTN (Chen et al. 2015), extracted, and concatenated with Phylosuite (Xiang et al. 2023). The multiple sequences were aligned using MAFFT v7.505 (Katoh and Standley 2013) with the auto strategy. The phylogenetic tree was constructed using Maximum likelihood (ML) method by IQ-TREE (Lanfear et al. 2020) software under the automatically selected GTR+F + R2 model.

## 3. Results

**Figure 2.**
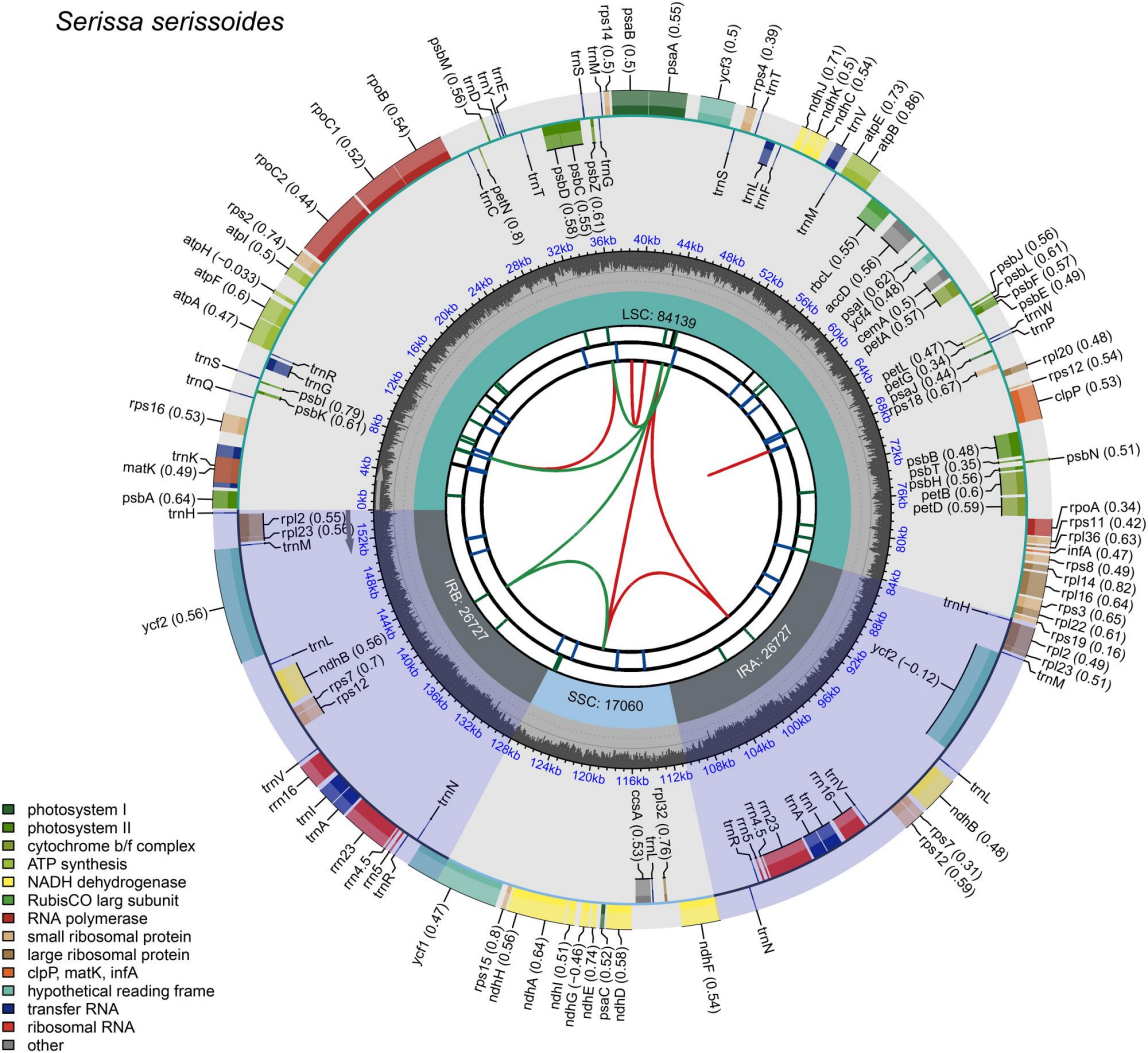
Circular map of the *S. serissoides* chloroplast genome. By default, the map consists of six concentric tracks arranged from the center outward. The innermost track represents dispersed repeats, followed by a track depicting long tandem repeats as short blue bars. The third track displays short tandem repeats (microsatellites) with colored bars. The fourth track delineates the genome’s structural regions, including SSC, LSC, IRa, and IRb. The fifth track illustrates the GC content along the genome, while the outermost track shows annotated genes, with codon usage bias optionally indicated in parentheses next to gene names. Genes are color-coded according to their functional categories, as explained in the legend at the bottom left, and are transcribed clockwise on the inner circle and counterclockwise on the outer circle.

The complete chloroplast genome *S. serissoides* of was 154,653 bp in length and exhibited a typical quadripartite structure (Figure 2). The large single-copy (LSC) region (84,139 bp) and a small single-copy (SSC) region (17,060 bp) were separated by a pair of inverted repeats (IR) regions (26,727 bp). The overall GC content is 37.84%, with variation observed among the different regions: 35.34% in the LSC region, 31.50% in the SSC region, and 42.77% in the IR region. The assembled genome exhibited sequencing coverage ranging from a minimum depth of 970× to a maximum of 4,768×, with an average depth of 3,305.73× (Figure S1). The chloroplast genome contained 130 unique genes (84 protein-coding genes, 38 tRNA genes, and 8 rRNA genes). including 6 protein-coding (*rpl*2, *rpl*123, *ycf*2, *ndh*B, *rps*7, *rps*12), 8 tRNA (*trn*H-GUG, *trn*I-CAU, *trn*L-CAA, *trn*V-GAC, trnI-GAU, trnA-UGC, trnR-ACG, trnN-GUU), and 4rRNA (*rrn*16S, *rrn*23S, *rrn*4.5S, *rrn*5S) genes duplicated in the IR regions. A total of 19 genes contain one intron, including 9 protein-coding genes (Figure S2). Two genes, *clp*P and *ycf*3, contain two introns (Figure S2). The *rps*12 gene is trans-spliced (Figure S3).

**Figure 3.**
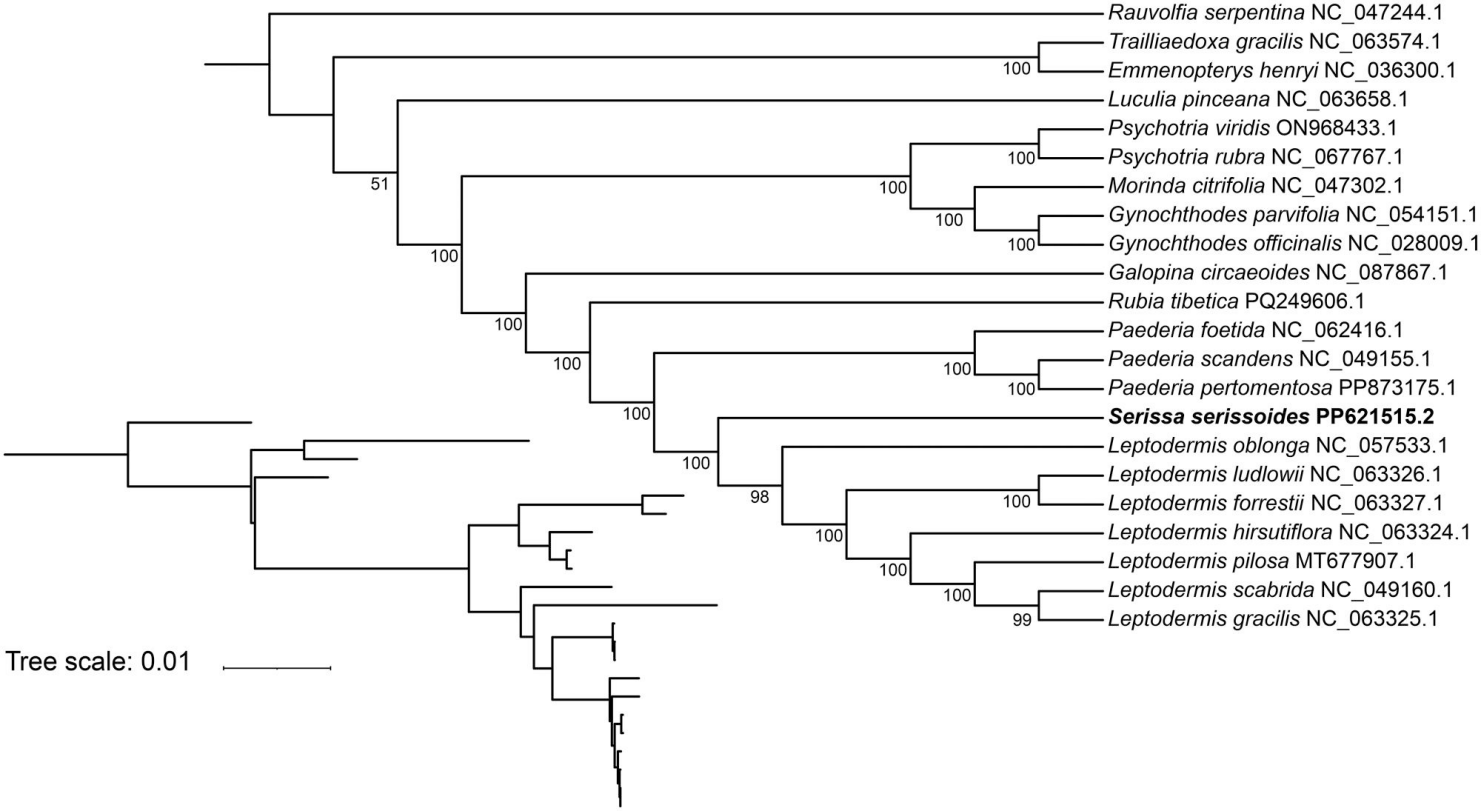
The phylogenetic tree was reconstructed using the ML method based on concatenated shared PCGs of the *S. serissoides* chloroplast genome. The species sequenced in this study is marked in bold. The following sequences were used: *Leptodermis forrestii* NC_063327.1 (Zhang, et al. 2021), *Leptodermis ludlowii* NC_063326.1 (Zhang, et al. 2021), *Leptodermis hirsutiflora* NC_063324.1 (Zhang, et al. 2021), *Leptodermis pilosa* MT677907.1, *Leptodermis oblonga* NC_057533.1, *Leptodermis gracilis* NC_063325.1 (Zhang, et al. 2021), *Paederia foetida* NC_062416.1, *Leptodermis scabrida* NC_049160.1 (Zhang et al. 2019), *Paederia scandens* NC_049155.1 (Li et al. 2019), *Paederia pertomentosa* PP873175.1, *Rubia tibetica* PQ249606.1 (Li et al. 2025), *Luculia pinceana* NC_063658.1, *Emmenopterys henryi* NC_036300.1 (Duan et al. 2017), *Trailliaedoxa gracilis* NC_063574.1 (Tan et al. 2023), *Galopina circaeoides* NC_087867.1 (Thureborn et al. 2024), *Psychotria rubra* NC_067767.1, *Psychotria viridis* ON968433.1 (Varani et al. 2022), *Morinda citrifolia* NC_047302.1 (Niu and Liu 2020), *Morinda officinalis* NC_028009.1, *Gynochthodes parvifolia* NC_054151.1 (Cai et al. 2020), and *Rauvolfia serpentina* NC_047244.1 as the outgroup.

The phylogenetic analysis demonstrated that *S. serissoides* is closely related to *Leptodermis* genus, with 100% guidance support (Figure 3). The phylogenetic tree structure is closely aligned with previous studies (Li et al. 2019; Zhang, et al. 2021).

## 4. Discussion and conclusions

*S. serissoides* belongs to the genus *Serissa* within the Rubiaceae. A chloroplast genome sequence of *S. serissoides* (accession number: ON014505.1) is available in NCBI; however, no corresponding publication has been released to date. In our study, we analyzed the chloroplast genome features of *S. serissoides*. Compared with the NCBI sequence (ON014505.1), our assembled genome differs by 52 bp in total length and by 0.36% in GC content, while the number of genes remains the same (Figure S4). Among the four regions, the largest difference in nucleotide length was found in the LSC region, with a difference of 40 bp. Collectively, these results demonstrate the overall conservation of the chloroplast genome of *S. serissoides*, with only slight discrepancies between assemblies.

Chloroplast genomes of Rubiaceae species are generally conserved in genome structure and gene content, with reported genome sizes ranging from approximately 151 to 161 kb (Ly et al. 2020; Safhi et al. 2024). In the present study, the chloroplast genome of *S. serissoides* showed a similar genome size, GC content, and gene composition to those reported for other Rubiaceae species (Li et al. 2019; Tan et al. 2023), indicating the conserved evolutionary characteristics of plastomes within this family. These conserved characteristics further support the application of chloroplast genomes as effective molecular resources for species identification and phylogenetic studies in Rubiaceae.

Although some taxonomic studies have treated *S. japonica* as a distinct species(NAM, et al. 2025; Zhang, et al. 2021), it is currently regarded as a synonym of *S. serissoides* in widely accepted taxonomic databases such as the Plants of the World Online (POWO) (https://powo.science.kew.org/taxon/urn:lsid:ipni.org:names:766333-1). This taxonomic inconsistency highlights the need for reliable molecular markers to clarify species boundaries within the genus. However, no publicly available chloroplast genome sequence or other genomic data for *S. japonica* have been reported to date, limiting comparative genomic analyses and the development and validation of species-specific molecular markers. Future studies incorporating genomic data from *S. japonica*, together with chloroplast, mitochondrial, and nuclear genomes, will help resolve the taxonomic status of *Serissa* and provide a more comprehensive understanding of its evolutionary relationships.

## Supplementary Material

Supplemental Material

Supplemental Material

Supplemental Material

Supplemental Material

## Data Availability

The genome sequence data that support the findings of this study are openly available in GenBank of NCBI at (https://www.ncbi.nlm.nih.gov/) under the accession number PP621515.2. The associated BioProject, SRA, and Bio-Sample numbers are PRJNA1097325, SRR28581776, and SAMN40869391, respectively.
