# Supplementary figures and images for: The complete chloroplast genome sequence of the medicinally important plant Serissa serissoides (DC.) Druce and phylogenetic analysis
Source: Mitochondrial DNA B Resour. 2026 Jul 27;11(8):967–71. doi: 10.1080/23802359.2026.2706902 (PMC13410745; doi:10.1080/23802359.2026.2706902)

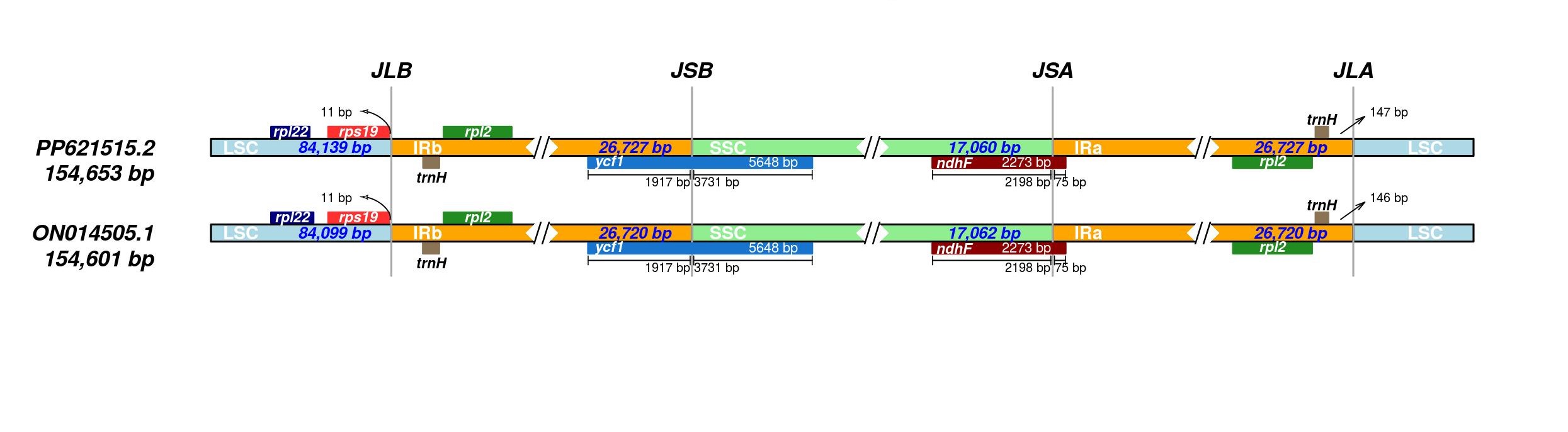

Supplement: Supplemental Material [file TMDN_A_2706902_SM5129.jpg]

# Trans-splicing Genes

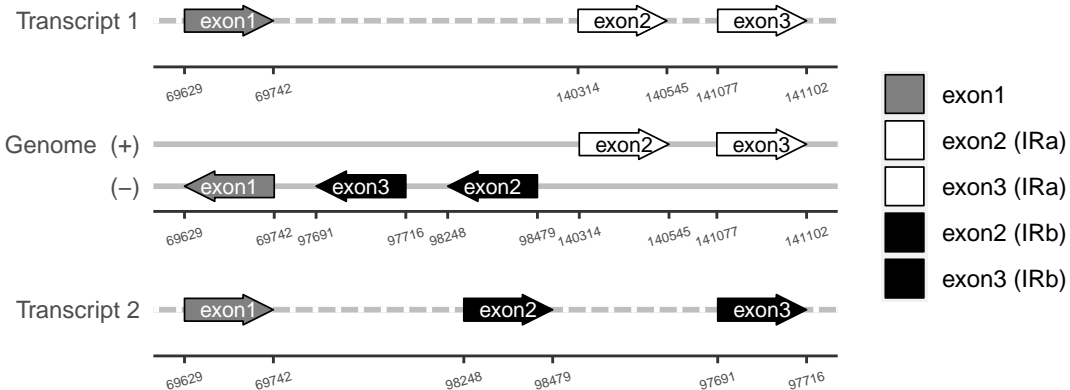

Supplement: Supplemental Material [file TMDN_A_2706902_SM5128.pdf]

# Cis-splicing Genes

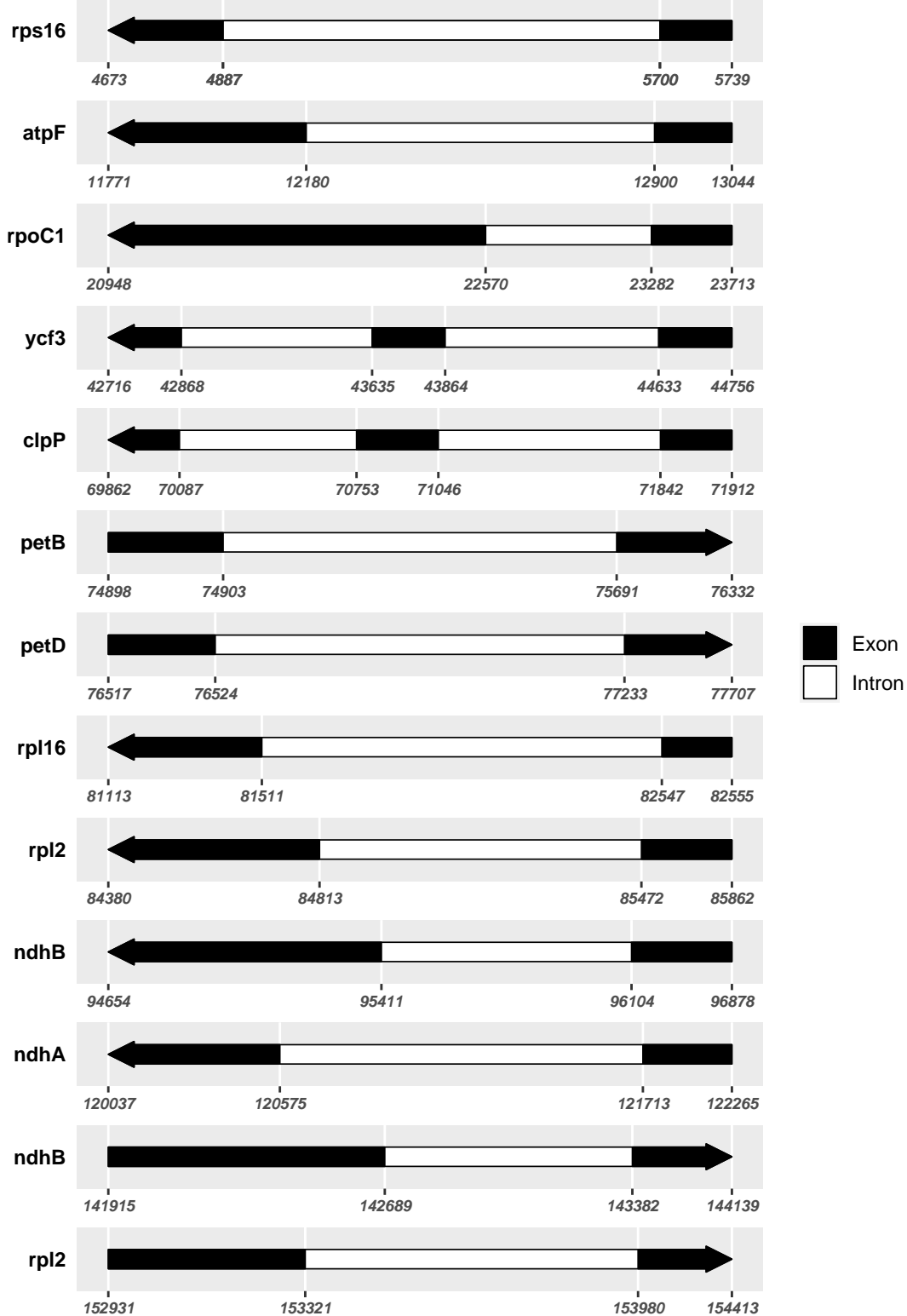

Supplement: Supplemental Material [file TMDN_A_2706902_SM5127.pdf]

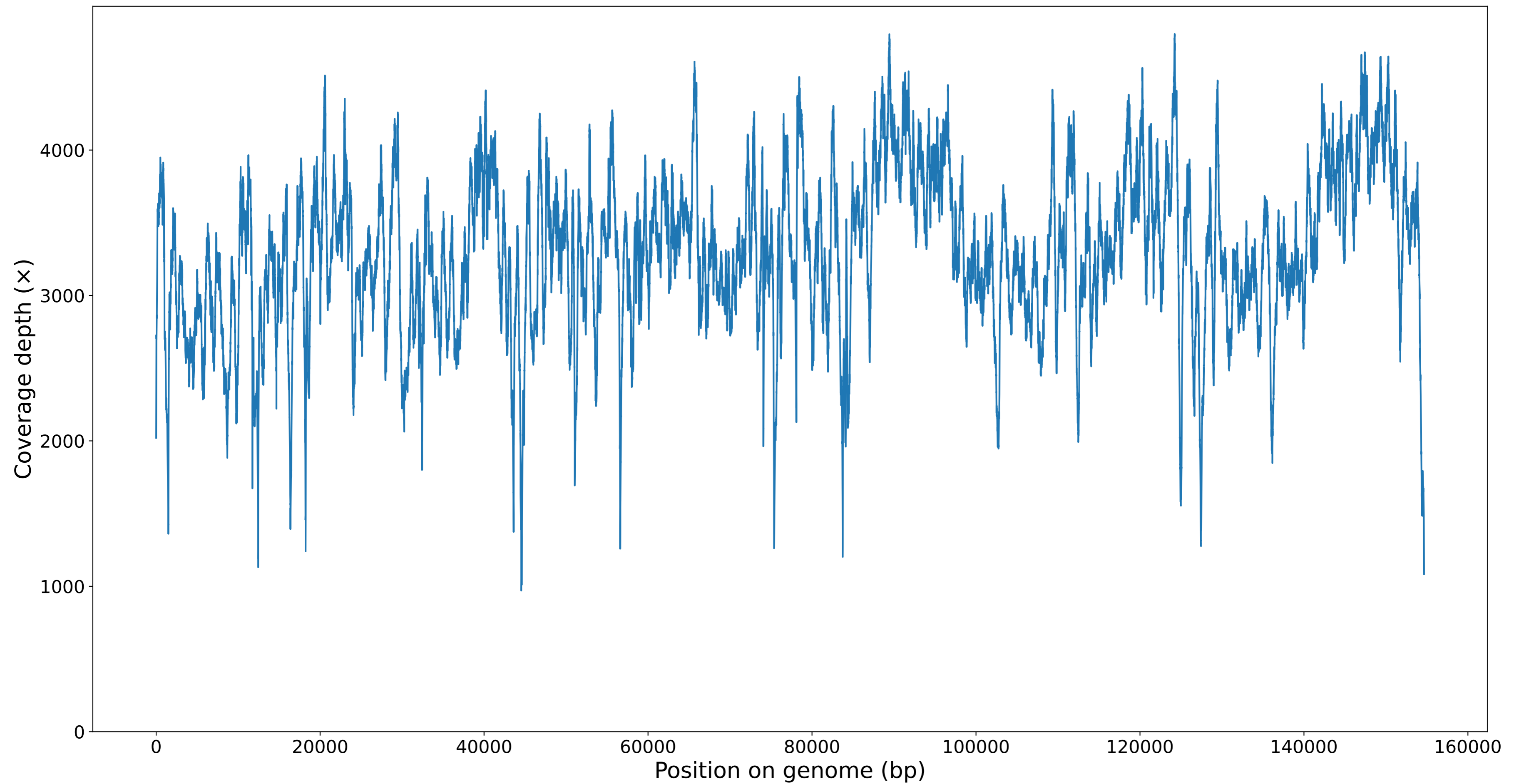

(1) Genome length: 154,653 bp    (2) Average depth: 3305.73x    (3) Maximal depth: 4798x    (4) Minimal depth: 970x

Supplement: Supplemental Material [file TMDN_A_2706902_SM5126.pdf]
